# Early Growth Response Genes Signaling Supports Strong Paracrine Capability of Mesenchymal Stem Cells

**DOI:** 10.1155/2012/428403

**Published:** 2012-12-06

**Authors:** Kenichi Tamama, Dominique J. Barbeau

**Affiliations:** Department of Pathology, School of Medicine, University of Pittsburgh, Pittsburgh, PA 15261, USA

## Abstract

MSCs provide a promising method for cell therapy through their wound healing and tissue regenerative properties. Originally, MSCs' role in wound healing was thought to be tied to their multipotency, but it is now accepted that MSCs mediate the healing process through their strong paracrine capability. EGF was shown to facilitate *in vitro* expansion of MSCs without altering multipotency. Our previous data suggest that the molecular machinery underlying MSCs' strong paracrine capability lies downstream of EGFR signaling, and we focus on transcription factors EGR1 and EGR2. Evidence suggests that EGR1 regulates angiogenic and fibrogenic factor production in MSCs, and an EGFR-EGR1-EGFR ligands autocrine loop is one of the underlying mechanisms supporting their strong paracrine machinery through EGR1. EGR2 appears to regulate the expression of immunomodulatory molecules. Chronic nonhealing wounds are ischemic, inflammatory, and often fibrotic, and the hypoxic micro-environment of these wounds may compromise MSCs' wound healing properties *in vivo* by upregulating the EGR1's fibrogenic effects and downregulating the EGR2's immuno-modulatory effects. Thus, these transcription factors can be potential targets in the optimization of cell-based therapies. Further study *in vitro* is required to understand MSCs' paracrine machinery and to optimize it as a tool for effective cell-based therapies.

## 1. Overview of MSCs

 Adult bone marrow multipotential stromal cells or mesenchymal stem cells (MSCs) are multipotent cells capable of differentiating into multiple cell lineages, such as osteocytes, adipocytes, and chondrocytes [1–7]. Because of their strong tissue regenerative, wound repair, and immunomodulatory effects, cell therapy with MSCs is highly promising against various diseases in the fields of regenerative medicine and immunology [8–15]. 

 MSC-based therapeutics was shown to accelerate the wound repair process in various animal models and pilot clinical studies including limb ischemia and coronary arterial diseases [8–14, 16–19]. However, beneficial results of stem/progenitor cell therapeutics in initial small-scale clinical studies have not been reproduced by subsequent randomized controlled trials, strongly indicating *the urgent needs of further optimization of cell-based therapy *[20]. 

 Initially, these cells were simply viewed as cellular blocks to resupply the regenerating and repairing tissues through their multidifferentiation potential; however, it is now widely accepted that MSCs' strong paracrine capability of various bioactive molecules such as vascular endothelial growth factor (VEGF) or indoleamine dioxygenase-1 (IDO1) plays a key role in MSC-based therapeutics actions [8, 15, 21–23]. In effect, MSCs, which reside within the perivascular space [24], can be viewed as paracrine delivery vehicles. Understanding of the molecular mechanism of the strong paracrine machinery of MSCs could lead to the identification of novel therapeutic targets and maximization of immuno-modulating, wound healing, and tissue regenerating effects of MSC-based therapeutics [25]. 

## 2. Roles of Epidermal Growth Factor Receptor Signaling in MSCs

*In vitro*, MSC expansion with animal component-free artificially-defined culture media is ideal for MSC preparation for clinical use to maximize the safety of MSC-based therapeutics [26–29]. Identification of key molecular factors for *in vitro* MSC expansion and understanding the molecular mechanism of MSCs' strong paracrine capability should provide key knowledge for *in vitro* MSC expansion without using any animal components while maintaining MSCs' paracrine capability. Moreover, advanced knowledge of molecular regulation of the angiogenic, mitogenic, fibrogenic, and immunomodulatory properties would allow for the MSC preparation of personalized properties to best fit the clinical needs of individual patients. 

 We previously showed that epidermal growth factor (EGF) could be used for *in vitro* MSC expansion without compromising their multidifferentiation potential [6, 30]. Moreover, EGF stimulation enhances the production of multiple growth factors and cytokines including VEGF, hepatocyte growth factor (HGF), Heparin-binding epidermal growth factor-like growth factor (HBEGF), and interleukin-6 (IL6) [25, 26]. These data strongly suggest that EGF can be used for *in vitro* MSC expansion and enhancement of their paracrine capability. 

 EGF receptor (EGFR) is a prototypal receptor tyrosine kinase widely expressed in many types of cells including MSCs [25, 26]. Upon binding of EGFR ligands such as EGF, HBEGF, or amphiregulin (AREG), EGFR undergoes dimerization and autophosphorylation through its intrinsic tyrosine kinase activity and activates numerous signaling pathways including the protein kinase C (PKC) pathway and the p42/44 mitogen-activated protein kinase (MAPK) pathway [6, 25, 26, 31]. 

 Based on our previous studies [6, 26], we speculated that the molecular machinery supporting MSCs' strong paracrine capability should be located downstream of EGFR signaling, and we analyzed the publicly available microarray database (GSE9451) to see whether transcription factors regulating the expression of growth factors and cytokines downstream of EGFR signaling are differentially expressed in human iliac bone marrow MSCs and human skin fibroblasts, another type of mesenchymal cells akin to MSCs but with reduced differentiation and paracrine capability. Our analysis showed that* early growth response genes-1, -2, and -3 (EGR1-3)* are expressed in MSCs at much higher levels than in fibroblasts (Table 1) [25]. High baseline expression of EGR1-3 in MSCs might reflect the activated state of MSCs in culture, as suggested by Caplan [32]. *EGRs* encode a zinc finger transcription factor (TF) whose activity is mainly regulated at the gene transcription level, and gene expression is upregulated in response to various growth factors and cytokines such as epidermal growth factor (EGF). Once induced, EGRs regulate the gene expression of various growth factors, cytokines, their cognate receptors, and other bioactive molecules [25, 33, 34]. 

## 3. Possible Roles of EGR1-3 in MSCs

 Although EGR1-3 has a highly conserved DNA-binding domain and share conserved zinc finger DNA-binding sequences [35], each EGR is regulated by distinct signaling pathways; for instance, PKC inhibitor bisindolylmaleimide I (BIM) weakly inhibits EGR1 induction [25], but it moderately inhibits EGR2 induction and totally abolishes EGR3 induction in MSCs (unpublished data). Moreover, each EGR confers functions that are largely distinctive from each other [36–39]. Among EGR1-3, EGR1 is the most studied, and its multiple roles have been proposed. For example, EGR1 is identified as one of the key molecules contributing to the development of atherosclerosis, intimal thickening after acute vascular injury, ischemic pathology, angiogenesis, allograft rejection, and cardiac hypertrophy [33, 40]. EGR1 could promote tumor progression, but at the same time, it could serve as a tumor suppressive or proapoptotic regulator [34, 41]. EGR2 is a key regulator of myelination in the nervous system and of hindbrain development [37, 42, 43]. It was also identified as a positive regulator of fibrosis development [36] and a negative regulator of T cell activation [44]. EGR3 was also identified to be critical in muscle spindle formation [45] and is a key regulator of endothelial cell activation by VEGF [39].

**Figure 1:**
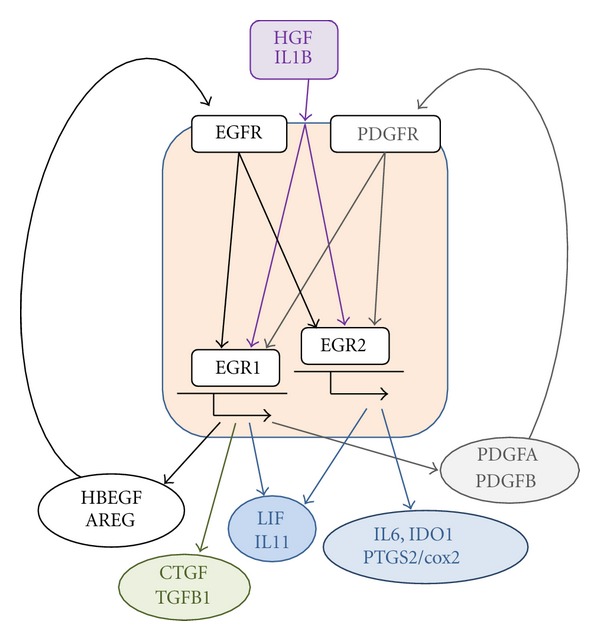
The roles of EGR1 and EGR2 signaling and autocrine loops with EGFR and PDGFR ligands in the production of select bioactive molecules in MSCs. (Abbreviations: CTGF, connective tissue growth factor; EGF, epidermal growth factor; EGFR, epidermal growth factor receptor; EGR, early growth response gene; HBEGF, heparin-binding epidermal growth factor-like growth factor; IDO1, indoleamine dioxygenase-1; IL1B, interleukin-1beta; IL6, interleukin-6; LIF, leukemia inhibitory factor; PDGFA, platelet-derived growth factor-A; PDGFB, platelet-derived growth factor-B; PTGS2/cox2, prostaglandin-endoperoxide synthase 2/cyclooxygenase-2; TGFB1, transforming growth factor-beta1; VEGFA, vascular endothelial growth factor-A).

 Biological roles of EGRs in MSCs have been addressed in only few studies including ours [25]. In this study, we showed that EGF receptor (EGFR) ligand is one of the strongest inducers of *EGR1* expression among the various growth factors and cytokines we evaluated. Upon EGF stimulation of MSCs, *EGR1* is strongly and transiently induced in a MAPK extracellular signal-regulated kinase (MEK) inhibitor sensitive manner. EGR1 signaling in turn upregulates growth factors and cytokines including EGFR ligands HBEGF and AREG in MSCs. Those data suggest the presence of an autocrine loop with an EGFR-EGR1-HBEGF/AREG axis. Moreover, HBEGF inhibitor CRM197 decreases the expression of *AREG, VEGF, leukemia inhibitory factor (LIF)*, and *interleukin 11 (IL11)* induced by a PKC activator phorbol ester, presumably by inhibiting HBEGF in the autocrine loop. Thus, even though binding of EGFR ligands to EGFR will eventually cause downregulation of EGFR and shutoff of the EGFR signaling [31], EGR1 still functions as a convergence point for multiple signaling pathways, and the EGFR-EGR1 axis could serve as a molecular machinery supporting the strong paracrine capability of MSCs, at least for select growth factors and cytokines described above (Figure 1). 

 Another possible autocrine loop exists involving platelet derived growth factor (PDGF), as both PDGF-AA and PDGF-BB upregulate *EGR1*, and gene expression of *PDGFA* and *PDGFB* is dependent on EGR1 (Figure 1) [25]. But both PDGF-AA and PDGF-BB do not induce EGR1 as strongly as EGF, and PDGFA and PDGFB expressions are not as high as HBEGF. Thus, we speculate that the PDGF-EGR1 autocrine loop is not as strong a contributor to MSCs' paracrine machinery as the EGFR-EGR1 autocrine loop. 

 Besides *HBEGF, AREG, PDGFA*, and *PDGFB*, our published and unpublished data also showed that EGR1 regulates gene expression of* connective tissue growth factor (CTGF) *and *transforming growth factor-beta 1 (TGFB1)* in MSCs [25]. Although HBEGF, AREG, PDGFA, PDGFB, and CTGF could promote angiogenesis and mitogenesis, these factors could enhance fibrogenesis in the presence of TGFB1 [46, 47], and thus, this group of molecules can be categorized as fibrogenic as well [48–51]. This is in agreement with a recent study showing that EGR1 was also identified as a key factor of fibrogenesis in dermal fibroblasts of patients with scleroderma and systemic sclerosis [52]. In other cell types, EGR1 has been reported to regulate various growth factors and cytokines other than the ones covered in this study [25]; and therefore, we speculate that the EGFR-EGR1 axis probably regulates expression of these factors in MSCs as well. 

 We are accumulating data about the roles of EGR2 in MSCs. Our unpublished data showed that EGR2 signaling appears to regulate expression of the molecules including *interleukin-6 (IL6), IDO1, LIF, *and* prostaglandin endoperoxide synthase 2/cyclooxygenase-2 (PTGS2/cox2),* all of which mediate immunomodulatory properties of MSCs (Figure 1) [15]. The role of EGR3 signaling in MSCs is unclear at that moment. 

 In our TRANSFAC database study, EGR1-3 have multiple consensus EGR-binding elements in their promoters (data not shown) and regulate expression of each other positively as well as negatively in a cell-type specific manner [53]. Indeed, direct EGR1 binding was observed in the putative promoters of *EGR1-3 *in the ENCODE database [54–56] available in the UCSC Genome Browser [57], and our preliminary results show direct EGR1 binding to the putative promoters of *EGR1-3 *by chromatin immunoprecipitation quantitative PCR (ChIP-qPCR) assays (data not shown). Moreover, EGR3 inhibition by siRNA increases the *EGR1* induction (data not shown). These data indicate the possible presence of the interaction among EGR1-3 in human MSCs. 

 These findings are, overall, distinct from the results obtained in other types of cells or organs outlined above. Based on our recent data, we speculate that EGRs are key molecular switches regulating the fibrogenic, angiogenic, and immunomodulatory properties of MSCs, and we could target EGR1 and EGR2 to maximize the beneficial effects of MSC-based therapeutics for therapies against various diseases including, but not limited to, chronic nonhealing wounds, ischemic diseases, and immune-mediated diseases [32].

 Various other stimuli could induce *EGR1-3 *and alter EGR signaling. For example, hepatocyte growth factor (HGF) and interleukin-1beta (IL1B) are strong inducers of EGR1-3 in MSCs [25]. Since these signaling molecules are also involved in wound repair and tissue regeneration [58, 59], they might function to augment EGR signaling in MSCs in wound microenvironments (Figure 1).

## 4. Hypoxic Microenvironments and EGR Signaling

 Wound repair and tissue regeneration play an indispensable role for humans to maintain life. It is also regarded as one of the most complicated biological processes involving various types of cells and bioactive molecules acting in a sophisticated fashion. The normal wound healing process occurs in three distinct, but overlapping stages: inflammation, new tissue formation, and remodeling [58], and any arrests in these processes lead to the formation of chronic nonhealing wounds. 

 Vascular complications can be the cause of wounds such as ischemic coronary diseases, as well as the direct result of injury or tissue destruction itself. The resultant ischemia is one of the main contributing factors to the arrest of the wound repair and tissue regeneration processes, since the limited supply of oxygen and other nutrients compromises cellular functions in the injured sites and impairs these processes [60]. Therapeutic angiogenesis restores the blood supply to these ischemic lesions and promotes wound repair and tissue regeneration. Local administrations of single angiogenic factors such as VEGF showed only limited benefit [61], suggesting that an administration of multiple growth factors and cytokines, rather than a single-specific growth factor, is required to attain functional vasculatures through neoangiogenesis [62]. MSCs produce multiple growth factors and cytokines in a coordinated manner in response to environmental cues; thus, MSC-based therapeutics could be one promising solution.

The microenvironments in nonhealing wounds, which require therapeutic interventions such as MSC-based therapeutics for healing, are largely hypoxic due to the compromised blood supply and inadequate angiogenesis [60]. Hypoxia itself activates various intracellular signaling in hypoxia inducible factor (HIF)-dependent and HIF-independent manners [63]. HIF is a master transcription factor regulating the expression of hundreds of genes through binding to HIF response element (HRE) in response to hypoxia. HIF consists of the constitutively expressed *β*-subunit (HIF-1*β*) and the regulatory *α*-subunit (HIF-1*α* and HIF-2*α*), which is stabilized in response to hypoxia. In contrast to ubiquitously expressed HIF-1*α*, the expression of HIF-2*α* is restricted to certain cell types such as vascular endothelial cells and is less characterized than HIF-1*α* [64, 65]. MSCs express HIF-2*α* in addition to HIF-1*α* [66, 67]. 

 We and others previously published the effects of hypoxia or hypoxic priming in MSC survival, the angiogenic factor production by MSCs, and *in vitro* MSC expansion [66, 68–75], but the molecular mechanisms of hypoxia-mediated altered EGR signaling have not been studied except for EGR1, which was reported to be upregulated by hypoxia in glioblastoma cells, monocytes, and hepatoma cells in a HIF-1-independent manner [76, 77]. Consistently, *EGR1* is upregulated by hypoxia in MSCs (unpublished data), and thus, EGR1 signaling in MSCs might be further enhanced in hypoxic microenvironments. Persistent and excessive inflammation is another pathophysiological feature of chronic nonhealing wounds [78, 79], and excessive inflammation also causes fibrosis and scar [80]. EGR1-mediated ECM formation should be a pivotal step in wound healing [81], but hypoxia could cause excessive activation of EGR1 signaling, which might further promote fibrosis formation in chronic wounds. 

 Our data also showed that hypoxic exposure decreases *EGR2* and *EGR3 *induction and expression of their target molecules in MSCs. Interestingly, HIF-2*α* appears to mediate the decrease of *EGR2* induction, at least partly; however, the role of HIF in the decrease of *EGR3* induction appears minimal if any. Based on these data, we speculate that hypoxia alters EGR2 and EGR3 signaling in MSCs and possibly reduces immunomodulatory properties of MSCs in the hypoxic microenvironments such as nonhealing wounds. MSCs' immunomodulatory properties might be possibly suboptimal through compromised *EGR2* induction in those microenvironments, which could lessen the overall wound repair and tissue regeneration properties of MSCs in the hypoxic microenvironments such as chronic nonhealing wounds because the reversal of persistent inflammation could promote their repair process [80].

## 5. Exosomes

 Recently, exosomes or microvesicles have been recognized as an alternative mechanism of intercellular communication [82]. Exosomes are membranous microvesicles (40–100 nm diameter) released into the extracellular space through exocytic fusion of multivesicular endosomes with the cell membrane [82]. In addition to protein and lipid components, RNAs are responsible for the exosome-mediated intercellular communication [83]. 

 Do exosomes mediate some of the MSCs' paracrine effects? MSCs were shown to be strong producers of exosomes [84]; and indeed, 10% of the total protein present in MSC-conditioned media was estimated to be derived from exosomes [85]. MSCs were shown to exert organ-protective effects via exosome [84, 86–89]. Moreover, MSC-derived exosomes seem to mediate some of MSCs' immunomodulatory effects [84]. However, some reports showed that it is the RNA components, not protein components, that mediate the action of MSC-derived exosomes [86, 89]. Thus, exosomes would mediate some of the MSCs' paracrine effects, but the precise roles of exosomes remain largely unknown at this point. 

## 6. Conclusion

 Understanding of MSCs' strong paracrine mechanism should provide molecular targets to optimally personalize the MSC preparations for individual patients. Our previous data suggested that EGR1 and EGR2 play key roles in the production of mitogenic, angiogenic, and immunomodulatory factors in MSCs. 

 EGR1 functions as a molecular switch of angiogenic, mitogenic, and fibrogenic factor production in MSCs. The EGFR-EGR1-HBEGF/AREG autocrine loop is one of the underlying mechanisms supporting their strong paracrine machinery through EGR1 signaling [25]. EGR2 appears to function as a molecular switch of immunomodulatory molecules in MSCs. Although stimulation with various growth factors and cytokines induces EGR1-3 *in vitro*, it might not necessarily reflect the MSCs' EGR response in harsh microenvironments* in vivo *such as ischemic lesions. Our data showed that hypoxic exposure lessens the induction of *EGR2* in cultured MSCs, suggesting that hypoxic microenvironments *in vivo *might compromise MSCs' immunomodulatory actions by reducing EGR2 signaling. 

 Through an understanding of their distinct roles in the regulation of various growth factors and cytokines, EGRs may provide a mechanism for altering the wound healing and tissue regenerative capabilities of MSCs through *in vitro* priming prior to patient treatment and/or molecular targeting *in vivo*, and thus, we propose that EGR1 and EGR2 can be potential molecular targets to maximize the paracrine capability of MSCs. Further *in vitro* studies to elucidate the molecular machinery underlying EGRs' paracrine capability are still needed to maximize the benefits of MSC-based cell therapies.

## Figures and Tables

**Table 1:** *EGR1-3* gene expression in human primary fibroblasts (FBs) and human primary mesenchymal stem cells (MSCs) from GEO database (GSE9451). *EGR1* data was published previously [25]. Gene expression was given in arbitrary units.

	FBs	MSCs	*P* value
*EGR1 *	325.5	1223.8	0.002
*EGR2 *	4.1	50.1	0.024
*EGR3 *	5.7	64.7	<0.001

## References

[1] Friedenstein AJ, Gorskaja UF, Kulagina NN (1976). Fibroblast precursors in normal and irradiated mouse hematopoietic organs. *Experimental Hematology*.

[2] Oswald J, Boxberger S, Jørgensen B (2004). Mesenchymal stem cells can be differentiated into endothelial cells in vitro. *Stem Cells*.

[3] Pittenger MF, Mackay AM, Beck SC (1999). Multilineage potential of adult human mesenchymal stem cells. *Science*.

[4] Prockop DJ (1997). Marrow stromal cells as stem cells for nonhematopoietic tissues. *Science*.

[5] Prockop DJ, Gregory CA, Spees JL (2003). One strategy for cell and gene therapy: harnessing the power of adult stem cells to repair tissues. *Proceedings of the National Academy of Sciences of the United States of America*.

[6] Tamama K, Fan VH, Griffith LG, Blair HC, Wells A (2006). Epidermal growth factor as a candidate for ex vivo expansion of bone marrow-derived mesenchymal stem cells. *Stem Cells*.

[7] Tamama K, Sen CK, Wells A (2008). Differentiation of bone marrow mesenchymal stem cells into the smooth muscle lineage by blocking ERK/MAPK signaling pathway. *Stem Cells and Development*.

[8] Kinnaird T, Stabile E, Burnett MS, Epstein SE (2004). Bone marrow-derived cells for enhancing collateral development: mechanisms, animal data, and initial clinical experiences. *Circulation Research*.

[9] Davani S, Marandin A, Mersin N (2003). Mesenchymal progenitor cells differentiate into an endothelial phenotype, enhance vascular density, and improve heart function in a rat cellular cardiomyoplasty model. *Circulation*.

[10] Amado LC, Saliaris AP, Schuleri KH (2005). Cardiac repair with intramyocardial injection of allogeneic mesenchymal stem cells after myocardial infarction. *Proceedings of the National Academy of Sciences of the United States of America*.

[11] Dai W, Hale SL, Martin BJ (2005). Allogeneic mesenchymal stem cell transplantation in postinfarcted rat myocardium: short- and long-term effects. *Circulation*.

[12] Pittenger MF, Martin BJ (2004). Mesenchymal stem cells and their potential as cardiac therapeutics. *Circulation Research*.

[13] Wu Y, Chen L, Scott PG, Tredget EE (2007). Mesenchymal stem cells enhance wound healing through differentiation and angiogenesis. *Stem Cells*.

[14] Sasaki M, Abe R, Fujita Y, Ando S, Inokuma D, Shimizu H (2008). Mesenchymal stem cells are recruited into wounded skin and contribute to wound repair by transdifferentiation into multiple skin cell type. *Journal of Immunology*.

[15] Uccelli A, Moretta L, Pistoia V (2008). Mesenchymal stem cells in health and disease. *Nature Reviews Immunology*.

[16] Mangi AA, Noiseux N, Kong D (2003). Mesenchymal stem cells modified with Akt prevent remodeling and restore performance of infarcted hearts. *Nature Medicine*.

[17] Giordano A, Galderisi U, Marino IR (2007). From the laboratory bench to the patient’s bedside: an update on clinical trials with mesenchymal stem cells. *Journal of Cellular Physiology*.

[18] Schuleri KH, Boyle AJ, Hare JM (2007). Mesenchymal stem cells for cardiac regenerative therapy. *Handbook of experimental pharmacology*.

[19] Falanga V, Iwamoto S, Chartier M (2007). Autologous bone marrow-derived cultured mesenchymal stem cells delivered in a fibrin spray accelerate healing in murine and human cutaneous wounds. *Tissue Engineering*.

[20] Tongers J, Losordo DW, Landmesser U (2011). Stem and progenitor cell-based therapy in ischaemic heart disease: promise, uncertainties, and challenges. *European Heart Journal*.

[21] Chen L, Tredget EE, Wu PYG, Wu Y, Wu Y (2008). Paracrine factors of mesenchymal stem cells recruit macrophages and endothelial lineage cells and enhance wound healing. *PLoS ONE*.

[22] Schinköthe T, Bloch W, Schmidt A (2008). In vitro secreting profile of human mesenchymal stem cells. *Stem Cells and Development*.

[23] Shimada IS, Spees JL (2011). Stem and progenitor cells for neurological repair: minor issues, major hurdles, and exciting opportunities for paracrine-based therapeutics. *Journal of Cellular Biochemistry*.

[24] Crisan M, Yap S, Casteilla L (2008). A perivascular origin for mesenchymal stem cells in multiple human organs. *Cell Stem Cell*.

[25] Kerpedjieva SS, Kim DS, Barbeau DJ, Tamama K (2012). EGFR ligands drive multipotential stromal cells to produce multiple growth factors and cytokines via early growth response-1. *Stem Cells and Development*.

[26] Tamama K, Kawasaki H, Wells A (2010). Epidermal growth factor (EGF) treatment on multipotential stromal cells (MSCs). Possible enhancement of therapeutic potential of MSC. *Journal of biomedicine & biotechnology*.

[27] Agata H, Watanabe N, Ishii Y (2009). Feasibility and efficacy of bone tissue engineering using human bone marrow stromal cells cultivated in serum-free conditions. *Biochemical and Biophysical Research Communications*.

[28] Ng F, Boucher S, Koh S (2008). PDGF, TGF-beta, and FGF signaling is important for differentiation and growth of mesenchymal stem cells (MSCs): transcriptional profiling can identify markers and signaling pathways important in differentiation of MSCs into adipogenic, chondrogenic, and osteogenic lineages. *Blood*.

[29] Sensebé L, Krampera M, Schrezenmeier H, Bourin P, Giordano R (2010). Mesenchymal stem cells for clinical application. *Vox Sanguinis*.

[30] Fan VH, Tamama K, Au A (2007). Tethered epidermal growth factor provides a survival advantage to mesenchymal stem cells. *Stem Cells*.

[31] Wells A (1999). EGF receptor. *International Journal of Biochemistry and Cell Biology*.

[32] Caplan AI (2009). Why are MSCs therapeutic? New data: new insight. *Journal of Pathology*.

[33] Silverman ES, Collins T (1999). Pathways of Egr-1-mediated gene transcription in vascular biology. *American Journal of Pathology*.

[34] Thiel G, Cibelli G (2002). Regulation of life and death by the zinc finger transcription factor Egr-1. *Journal of Cellular Physiology*.

[35] Christy B, Nathans D (1989). DNA binding site of the growth factor-inducible protein Zif268. *Proceedings of the National Academy of Sciences of the United States of America*.

[36] Fang F, Ooka K, Bhattachyya S (2011). The early growth response gene Egr2 (alias Krox20) is a novel transcriptional target of transforming growth factor-*β* that is up-regulated in systemic sclerosis and mediates profibrotic responses. *American Journal of Pathology*.

[37] Le N, Nagarajan R, Wang JYT, Araki T, Schmidt RE, Milbrandt J (2005). Analysis of congenital hypomyelinating Egr2Lo/Lo nerves identifies Sox2 as an inhibitor of Schwann cell differentiation and myelination. *Proceedings of the National Academy of Sciences of the United States of America*.

[38] Lee SL, Sadovsky Y, Swirnoff AH (1996). Luteinizing hormone deficiency and female infertility in mice lacking the transcription factor NGFI-A (Egr-1). *Science*.

[39] Suehiro JI, Hamakubo T, Kodama T, Aird WC, Minami T (2010). Vascular endothelial growth factor activation of endothelial cells is mediated by early growth response-3. *Blood*.

[40] Khachigian LM (2006). Early growth response-1 in cardiovascular pathobiology. *Circulation Research*.

[41] Adamson ED, Mercola D (2002). Egr1 transcription factor: multiple roles in prostate tumor cell growth and survival. *Tumor Biology*.

[42] Street VA, Bennett CL, Goldy JD (2003). Mutation of a putative protein degradation gene LITAF/SIMPLE in Charcot-Marie-Tooth disease 1C. *Neurology*.

[43] Warner LE, Mancias P, Butler IJ (1998). Mutations in the early growth response 2 (EGR2) gene are associated with hereditary myelinopathies. *Nature Genetics*.

[44] Safford M, Collins S, Lutz MA (2005). Egr-2 and Egr-3 are negative regulators of T cell activation. *Nature Immunology*.

[45] Tourtellotte WG, Milbrandt J (1998). Sensory ataxia and muscle spindle agenesis in mice lacking the transcription factor Egr3. *Nature Genetics*.

[46] Bonner JC (2004). Regulation of PDGF and its receptors in fibrotic diseases. *Cytokine and Growth Factor Reviews*.

[47] Leask A (2010). Potential therapeutic targets for cardiac fibrosis: TGF*β*, angiotensin, endothelin, CCN2, and PDGF, partners in fibroblast activation. *Circulation Research*.

[48] Barrientos S, Stojadinovic O, Golinko MS, Brem H, Tomic-Canic M (2008). Growth factors and cytokines in wound healing. *Wound Repair and Regeneration*.

[49] Beyer C, Distler JH, Distler O (2010). Are tyrosine kinase inhibitors promising for the treatment of systemic sclerosis and other fibrotic diseases?. *Swiss Medical Weekly*.

[50] Chen J, Chen JK, Nagai K (2012). EGFR signaling promotes TGFbeta-dependent renal fibrosis. *Journal of the American Society of Nephrology*.

[51] Ingram JL, Bonner JC (2006). EGF and PDGF receptor tyrosine kinases as therapeutic targets for chronic lung diseases. *Current Molecular Medicine*.

[52] Bhattacharyya S, Sargent JL, Du P (2011). Egr-1 induces a profibrotic injury/repair gene program associated with systemic sclerosis. *PLoS ONE*.

[53] Kumbrink J, Kirsch KH, Johnson JP (2010). EGR1, EGR2, and EGR3 activate the expression of their coregulator NAB2 establishing a negative feedback loop in cells of neuroectodermal and epithelial origin. *Journal of Cellular Biochemistry*.

[54] Feingold EA, Good PJ, Guyer MS (2004). The ENCODE (ENCyclopedia of DNA Elements) Project. *Science*.

[55] Feingold EA, Good PJ, Guyer MS (2011). A user’s guide to the encyclopedia of DNA elements (encode). *PLoS Biology*.

[56] Rosenbloom KR, Dreszer TR, Long JC (2012). ENCODE whole-genome data in the UCSC Genome Browser: update 2012. *Nucleic Acids Research*.

[57] Meyer LR, Zweig AS, Hinrichs AS The UCSC Genome Browser database: extensions and updates 2013.

[58] Gurtner GC, Werner S, Barrandon Y, Longaker MT (2008). Wound repair and regeneration. *Nature*.

[59] Martin P (1997). Wound healing—aiming for perfect skin regeneration. *Science*.

[60] Sen CK (2009). Wound healing essentials: let there be oxygen. *Wound Repair and Regeneration*.

[61] Dvorak HF (2005). Angiogenesis: update 2005. *Journal of Thrombosis and Haemostasis*.

[62] Tamama K, Kerpedjieva SS (2012). Acceleration of wound healing by multiple growth factors and cytokines secreted from multipotential stromal cells/mesenchymal stem cells (MSCs). *Advances in Wound Care*.

[63] Simon MC, Keith B (2008). The role of oxygen availability in embryonic development and stem cell function. *Nature Reviews Molecular Cell Biology*.

[64] Tian H, McKnight SL, Russell DW (1997). Endothelial PAS domain protein 1 (EPAS1), a transcription factor selectively expressed in endothelial cells. *Genes and Development*.

[65] Wiesener MS, Jürgensen JS, Rosenberger C (2003). Widespread hypoxia-inducible expression of HIF-2alpha in distinct cell populations of different organs. *The FASEB Journal*.

[66] Tamama K, Kawasaki H, Kerpedjieva SS, Guan J, Ganju RK, Sen CK (2011). Differential roles of hypoxia inducible factor subunits in multipotential stromal cells under hypoxic condition. *Journal of Cellular Biochemistry*.

[67] Tamama K, Kim D, Kerpedjieva SS, Vordermark D Molecular mechanisms of hypoxia-mediated enhanced in vitro expansion, augmented self-renewal, and increased therapeutic potential of mesenchymal stem cells. *Hypoxia: Causes, Types and Management*.

[68] Crisostomo PR, Wang Y, Markel TA, Wang M, Lahm T, Meldrum DR (2008). Human mesenchymal stem cells stimulated by TNF-*α*, LPS, or hypoxia produce growth factors by an NF*κ*B- but not JNK-dependent mechanism. *American Journal of Physiology*.

[69] Das R, Jahr H, van Osch GJ, Farrell E (2010). The role of hypoxia in bone marrow-derived mesenchymal stem cells: considerations for regenerative medicine approaches. *Tissue engineering B*.

[70] Mylotte LA, Duffy AM, Murphy M (2008). Metabolic flexibility permits mesenchymal stem cell survival in an ischemic environment. *Stem Cells*.

[71] Okuyama H, Krishnamachary B, Zhou YF, Nagasawa H, Bosch-Marce M, Semenza GL (2006). Expression of vascular endothelial growth factor receptor 1 in bone marrow-derived mesenchymal cells is dependent on hypoxia-inducible factor 1. *The Journal of Biological Chemistry*.

[72] Rosová I, Dao M, Capoccia B, Link D, Nolta JA (2008). Hypoxic preconditioning results in increased motility and improved therapeutic potential of human mesenchymal stem cells. *Stem Cells*.

[73] Song SW, Chang W, Song BW (2009). Integrin-linked kinase is required in hypoxic mesenchymal stem cells for strengthening cell adhesion to ischemic myocardium. *Stem Cells*.

[74] Xu R, Chen J, Cong X, Hu S, Chen X (2008). Lovastatin protects mesenchymal stem cells against hypoxia- and serum deprivation-induced apoptosis by activation of PI3K/Akt and ERK1/2. *Journal of Cellular Biochemistry*.

[75] Zhu W, Chen J, Cong X, Hu S, Chen X (2006). Hypoxia and serum deprivation-induced apoptosis in mesenchymal stem cells. *Stem Cells*.

[76] Rong Y, Hu F, Huang R (2006). Early growth response gene-1 regulates hypoxia-induced expression of tissue factor in glioblastoma multiforme through hypoxia-inducible factor-1-independent mechanisms. *Cancer Research*.

[77] Yan SF, Lu J, Zou YS (1999). Hypoxia-associated induction of early growth response-1 gene expression. *The Journal of Biological Chemistry*.

[78] Pierce GF (2001). Inflammation in nonhealing diabetic wounds: the space-time continuum does matter. *American Journal of Pathology*.

[79] Schaffer M, Witte M, Becker HD (2002). Models to study ischemia in chronic wounds. *The International Journal of Lower Extremity Wounds*.

[80] Willenborg S, Knipper J, Ranjan R, Krieg T, Eming SA, Sen CK (2010). Chronic Wounds and Inflammation. *Wound Healing Society Year Book (WHSYB)—Advances in Wound Care*.

[81] Wu M, Melichian DS, De La Garza M (2009). Essential roles for early growth response transcription factor Egr-1 in tissue fibrosis and wound healing. *American Journal of Pathology*.

[82] Février B, Raposo G (2004). Exosomes: Endosomal-derived vesicles shipping extracellular messages. *Current Opinion in Cell Biology*.

[83] Valadi H, Ekström K, Bossios A, Sjöstrand M, Lee JJ, Lötvall JO (2007). Exosome-mediated transfer of mRNAs and microRNAs is a novel mechanism of genetic exchange between cells. *Nature Cell Biology*.

[84] Yeo RW, Lai RC, Zhang B Mesenchymal stem cell: an efficient mass producer of exosomes for drug delivery.

[85] Lai RC, Chen TS, Lim SK (2011). Mesenchymal stem cell exosome: a novel stem cell-based therapy for cardiovascular disease. *Regenerative Medicine*.

[86] Bruno S, Grange C, Deregibus MC (2009). Mesenchymal stem cell-derived microvesicles protect against acute tubular injury. *Journal of the American Society of Nephrology*.

[87] Collino F, Deregibus MC, Bruno S (2010). Microvesicles derived from adult human bone marrow and tissue specific mesenchymal stem cells shuttle selected pattern of miRNAs. *PLoS ONE*.

[88] Lai RC, Arslan F, Lee MM (2010). Exosome secreted by MSC reduces myocardial ischemia/reperfusion injury. *Stem Cell Research*.

[89] Tomasoni S, Longaretti L, Rota C Transfer of growth factor receptor mRNA via exosomes unravels the regenerative effect of mesenchymal stem cells.

